# The association between teacher distress and student mental health outcomes: a cross-sectional study using data from the school mental health survey

**DOI:** 10.1186/s40359-024-02071-3

**Published:** 2024-10-23

**Authors:** Vanessa De Rubeis, Ruth Repchuck, Jillian Halladay, Katherine T. Cost, Lehana Thabane, Katholiki Georgiades

**Affiliations:** 1 Department of Psychiatry and Behavioural Neurosciences, McMaster University, 1280 Main St W, Hamilton, ON L8S 4K1 Canada; 2 Offord Centre for Child Studies, McMaster University, 1280 Main St W, Hamilton, ON L8S 4K1 Canada; 3 School of Nursing, McMaster University, 280 Main St W, Hamilton, ON L8S 4L8 Canada; 4 Peter Boris Centre for Addictions Research, McMaster University / St. Joseph’s Healthcare, Hamilton, ON Canada; 5 The Matilda Centre for Research in Mental Health and Substance Use, University of Sydney, Sydney, NSW Australia; 6 SickKids Research Institute, The Hospital for Sick Children, 686 Bay St, Toronto, ON M5G 0A4 Canada; 7 Department of Health Research Methods, Evidence, and Impact, McMaster University, 280 Main St W, Hamilton, ON L8S 4L8 Canada; 8 Research Institute of St Joes Hamilton, St Joseph’s Healthcare—Hamilton, Hamilton, ON Canada; 9 Faculty of Health Sciences, University of Johannesburg, Johannesburg, South Africa

**Keywords:** Student mental health, Teacher distress, Internalizing symptoms, Externalizing symptoms

## Abstract

**Background:**

Few studies have examined the inter-relationships between teacher and student mental health. We aimed to examine associations between teacher distress and student mental health difficulties and if student perceptions of school safety moderate these associations.

**Method:**

Data from 23,568 students in grades 6–12 and 1,478 teachers from 268 schools participating in the School Mental Health Surveys in Ontario, Canada, were used. Three-level (student, classroom, school) multivariable linear regression models were fit to examine associations between teacher distress and student internalizing and externalizing symptoms by elementary (grades 6–8) and secondary (grades 9–12) school. Statistical interactions were used to evaluate effect modification.

**Results:**

Small but statistically significant, positive associations were found between teacher distress and internalizing (*b* = 0.02; 95% CI [0.01, 0.04], *p* < 0.05) and externalizing symptoms (*b* = 0.03; 95% CI [0.01, 0.05], *p* < 0.001) among elementary students only. Student perceptions of school safety moderated the association between teacher distress and externalizing symptoms among elementary students, whereby the positive association was magnified among students reporting lower school safety.

**Conclusions:**

Findings from this study highlight the importance of concurrently addressing the mental health needs of educators and students. School safety represents a modifiable target for prevention and intervention efforts in schools that could serve to promote student mental health and mitigate potential risk factors in schools.

**Supplementary Information:**

The online version contains supplementary material available at 10.1186/s40359-024-02071-3.

## Introduction

As of 2014, 18–22% of children and youth aged 4 to 17 years in Ontario met *DSM-IV-TR* criteria for a mental disorder [1]. Childhood mental disorders have been associated with significant distress and impairment across numerous domains of functioning both concurrently and across the life course [2]. Characteristics and experiences within the school environment have the potential to shape student mental health, given the significant amount of time children and youth spend in school and with their teachers [3, 4]. Few studies have examined the inter-relationship between student and teacher mental health, although some evidence suggests positive associations [2, 5, 6].

About 46% of teachers experience daily stress while teaching, often related to feelings of burnout [10, 11]. Internationally, increased stress experienced by teachers is leading to high attrition rates [10]. Teacher distress or burnout may be influenced by several stressors operating within the school environment, including inadequate resources, large class sizes, lack of autonomy, increased workload, limited social and supervisory support, managing challenging student behaviours, and responding to students’ emotional and behavioural needs [6, 12]. This increased stress is important for teachers’ well-being but also may play an important role in the well-being of their students [2, 10, 13].

Few studies have examined associations between teacher distress and student mental health-related outcomes [2, 5, 14–17]. Feelings of teacher stress, distress, or burnout have been associated with lower student performance, well-being and mental health outcomes [14–17]. For instance, Harding et al. found that teacher well-being was positively associated with student well-being and negatively associated with psychological distress among secondary students [2]. Another study found that students in classrooms with a teacher who reported higher feelings of burnout had higher morning cortisol - a measure of physiological stress [5]. It is possible that feelings of distress among teachers may influence their capacity to maintain positive classroom environments or notice students in distress and respond effectively to support them [5]. Most of these studies were cross-sectional and used multivariable multilevel models but were limited by small sample sizes, convenience samples, low response rates [2, 5, 14], and/or did not specifically measure student mental health outcomes [16, 17]. These methodological characteristics limit generalizability and pose challenges when seeking to inform strategies to improve mental health in schools at a population level.

The school environment, including perceptions of school safety, may amplify or attenuate associations between teacher and student mental health. Existing evidence suggests that both teachers and students with higher perceptions of unsafe school environments report worse mental health [18–20]. This is likely due to a number of factors that contribute to perceptions of an unsafe school environment, including but not limited to, experiences of peer victimization and violence and limited adult supervision [18].

We used the social-ecological framework by Townsend and Foster [21] and the emotional contagion theory [22] to inform the rationale and objectives of this study. The social-ecological theory is a framework specific to the school setting used to explore students’ health behaviors. Foster and Townsend identified six levels of influence: student demographics, student intrapersonal, student interpersonal, school organization, school community, and macro-level organization [21]. This approach to understanding student mental health ensures that essential factors are identified that play crucial roles in student mental health. The social-ecological framework proposes that a student’s interpersonal relationships at school may affect behaviour, such as relationships between teachers and students [21]. This can be supplemented by applying the emotional contagion theory, which states people adopt similar emotions of those around them or in a shared environment [17, 22]. This emotional contagion theory has been further adapted to focus on stress, since experiences or feelings of stress may spread within social networks [23]. Further, the social-ecological theory can be used to inform how the school environment (e.g., school safety) may influence student outcomes, as these experiences may interact with, or influence, each level within the school setting. Using these theories, we hypothesized that teacher distress will be positively associated student mental health symptoms even after controlling for variables at different levels of influence. Given differences between elementary and secondary school settings, including the number of different teachers a student may have or available mental health resources and supports, we conducted separate analyses for each.

To the best of our knowledge, this is the first study to examine the association between teacher distress and student mental health outcomes using a large representative sample of elementary (grades 6–8) and secondary (grades 9–12) schools across the province of Ontario, Canada. Our study integrates multi-informant data from both teacher and student reported measures that are psychometrically sound and our statistical analyses adjust for a number of known covariates across multiple levels of the school environment (student, classroom, and school). The objectives of this study were:

1. to explore associations between teacher-reported distress and student-reported internalizing and externalizing mental health symptoms; and
2. to explore the extent to which student perceptions of school safety moderate these associations.

The findings from this study can be used to inform the development of student mental health prevention and intervention strategies and point to potential, modifiable targets for intervention efforts, such as school safety. This study also highlights the importance of concurrently considering and addressing the mental health needs of both teachers and students in school settings.

## Materials and methods

### Study design and data source

Data for this study comes from the 2014–2015 School Mental Health Survey (SMHS) [24] - a cross-sectional study that included 248 schools across Ontario, Canada. Schools were selected based on the sampling design of the Ontario Child Health Study, a companion study of the epidemiology of child and youth mental health in the province of Ontario [25]. Selected schools were no different from schools not selected on the basis of school type, school level, language, region, enrolment size, proportion of English Language Learners, standardized achievement levels, and sociodemographic characteristics derived from the National Household Survey and the Census. Among those schools selected, 60% agreed to participate. Once a school agreed to participate, all teachers and students in grades 6–8 were invited to participate. In secondary schools, a random sample of three classrooms per grade (grades 9 to 12) was selected, and students and teachers from those classes were invited to participate. In total, 31,124 students participated (student response rate was 62%) and 3,373 teachers from kindergarten to grade 12 (teacher response rate was 73.5%).

The sample for analyses in the present study included 23,568 students whose teachers completed the teacher survey and had complete mental health data (75.7% of all students completing the SMHS).

**Fig. 1:**
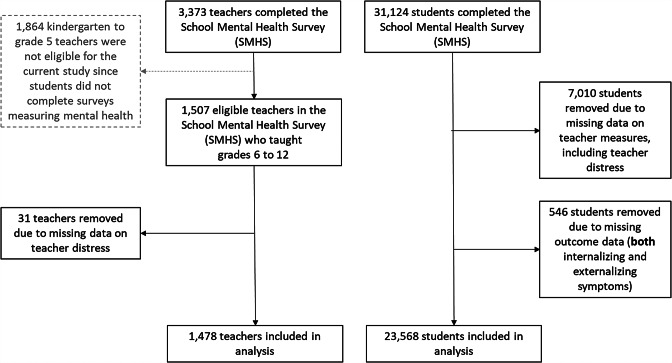
In total, 3,372 teachers completed the School Mental Health Survey (SMHS). Only teachers who taught grades 6 to 12 were eligible to be included (n=1,507). An additional 31 teachers who were missing data on the measure of teacher distress (independent variable), were removed. This left a total of 1,478 teachers eligible to be included in the current study. In total, 31,123 students completed the SMHS. 7,010 students were removed who had a teacher missing the teacher distress measure (independent variable). An additional 546 students were removed who were missing both outcome measures (internalizing and externalizing symptoms). This left a total of 23, 568 students eligible to be included in the current analysis

Among teachers, 1,509 were eligible to be included in this study since they taught grades 6 to 12. Kindergarten to grade 5 and special education teachers were not eligible to be included in this study since students in their classrooms did not complete corresponding surveys measuring their mental health. About 2.0% (*n = 31)* of teachers had missing responses on the psychological distress measures and were removed, resulting in a final sample of 1,478 teachers (Fig. 1).

### Student mental health outcomes (internalizing and externalizing symptoms)

#### Internalizing symptoms

To measure internalizing symptoms, a modified subset including nine items from the *Ontario Child Health Study Emotional Behavioural Scales* (OCHS-EBS) was used [26]. Students were asked about *feeling unhappy*, *sad or depressed*; *moody or irritable*; *having no pleasure from usual activities*; *feeling overtired or a lack of energy*; *feeling worthless or inferior*; *fearful or anxious*; *finding it hard to stop worrying*; *anxious or on edge*; or *nervous or tense*. They were asked to select the response that best describes them now or within the past six months. Response options included [1] *never or not true*, [2] *sometimes or somewhat true*, [3] *often or very true*. Scores were summed and ranged from a minimum of 9, indicating low internalizing symptoms, to 27, indicating high internalizing symptoms. Within the SMHS sample, the scale had high internal consistency among elementary (α = 0.89) and secondary students (α = 0.91) [9].

#### Externalizing symptoms

A modified OCHS-EBS scale with 13 items was used to measure externalizing symptoms [26]. Students were asked about *losing temper*; *arguing with adults*; *acting defiant or talking back to people*; *feeling angry or resentful*; *having feelings of wanting to get back at people*; *threatening to hurt people*; *physically attacking people*; *getting into fights*; *damaging schools or property*; *acting disobedient at school*; *using weapons when fighting*; *stealing things from places other than home*; and *having broken into someone else’s house*, *building*, * or car*. Participants were asked to select the response that best describes them now or within the past six months. Response options included [1] *never or not true*, [2] *sometimes or somewhat true*, [3] *often or very true.* Scores were summed and ranged from 13 to 39, with a higher score indicating higher externalizing symptoms. This scale has been found to have high internal consistency among elementary (α = 0.87) and secondary students (α = 0.88) [9].

### Independent variable

#### Teacher distress

Teacher distress was measured using the *Kessler Psychological Distress Scale (K6)*. This 6-item scale is widely used in epidemiological surveys as it is a brief tool that is both valid and reliable [27]. Teachers were asked, “During the past 30 days, how often do you feel the following: nervous; hopeless; restless or fidgety; depressed that nothing could cheer you up; that everything was an effort; worthless?”. They were given the following response options: [0] None of the time; [1] A little of the time; [2] Some of the time; [3] Most of the time; [4] All of the time. Possible scores ranged from 0 to 24. Within the SMHS, teacher distress had a Cronbach’s α of 0.86, indicating high internal consistency. Teacher distress was modelled at the classroom level (level 2).

### Individual/student-level variables

#### Gender

Students were asked to identify as [0] male or [1] female.

#### Age

Students were asked to report their age in years. The mean age for elementary students was 12.2 years (*SD* = 1.05), and for secondary students was 15.5 years (*SD* = 1.46).

*Race and cultural group.* Participants were asked, “Which category best describes your race or cultural group?”, with the option to select more than one category. To ensure a sufficient sample size within each category, racial and ethnic groups were collapsed into the following groupings: White (reference); East Asian, Southeast Asian, or South Asian (Asian); Black African, Black Caribbean, or Black Canadian or American (Black); or Other/Multiracial (including West Asian or Arab, Latin American, Central American, South American, Aboriginal/Native, Other, or Multiracial). White youth were chosen as the reference group as they represented the largest proportion of the sample.

#### Family assets

Family assets were measured using a modified version of the Family Affluence Scale [28, 29] from the *Health Behaviour in School-Aged Children Survey* [30]. Students were asked to report how many of the following their family owned: [1] a car, van, or truck; [2] a desktop computer or laptop; [3] a cell phone and a tablet; [4] an e-reader or iPad. Response options were on a 4-point scale: none, 1, 2, 3 or more. To reduce the number of variables entered into the analysis and to develop a standardized z-score, principal component analysis was used. A single component emerged, accounting for 45.5% of the variance.

#### School safety

To measure school safety, a scale was derived from the *Survey of Chicago Public Schools Elementary School Student Edition* [31]. This scale asked students to report how safe they felt in five school environments, including: hallways/stairwells; bathrooms/changes rooms; outside/around the school; on way to/from school, and in classes. Response options included: [1] not safe; [2] somewhat safe; [3] mostly safe; [4] very safe. Scores ranged from 5 to 20, with higher scores indicating higher feelings of school safety. Cronbach’s alpha for this scale was high (α = 0.86), indicating good internal consistency within the SMHS.

### Classroom-level variables

#### Classroom size

Teachers were asked to report how many students were enrolled in their class. Classroom size was treated as a categorical variable with response options [1] 1–5; [2] 16–20; [3] 21–25; [4] 26–30 (*reference)*; [5] ≥ 31.

### School level-variables

#### School socioeconomic status

Information was derived from the *National Household Survey 2011* [31] to measure school socioeconomic status. A weighted mean of median family income by postal code was used, whereby postal codes with more students were weighted more heavily than those with fewer students. An average for each school was estimated. Median family income is presented in increments of $1000.

#### School urbanicity

Depending on where schools were located, they were coded as urban [0] or rural [1] using the school postal code.

### Statistical analysis

All statistical analyses were conducted using SAS 9.4 [32]. To determine the association between teacher distress and student mental health outcomes (internalizing and externalizing symptoms), a three-level (school, classroom, student) multivariable linear regression model was fit for each outcome. Separate models were run for elementary (grades 6–8) and secondary schools (grades 9–12). Random intercepts were included in the model for classrooms and schools, and all remaining covariates were included as fixed effects. To determine model adequacy, model assumptions were explored (including linearity, homoskedasticity, and collinearity). PROC GLIMMIX was used to calculate unstandardized coefficients (b) and 95% confidence intervals (CIs). A second model was run where the main effect for school safety and the interaction term for school safety and teacher distress were added to understand how this variable modified associations. A complete case analysis was conducted as all variables had less than 5% missing. Standardized models were also run using the PROC STDIZE function in SAS (Appendix Tables A2 and A3).

## Results

Table 1 describes the characteristics of the study sample, by elementary (*n* = 14,665) and secondary students (*n* = 8,903). In both elementary and secondary schools, approximately equal number of male (52%) and female students (47%) participated. Most teachers were female in both elementary (64.1%) and secondary schools (58.9%) (Table A1). Among elementary students, the average scores for internalizing and externalizing symptoms, were 13.3 and 15.8, respectively. Among secondary students, average scores were slightly higher, 14.8 for internalizing and 16.0 for externalizing symptoms. The average score for teacher distress was 4.1 in elementary and 3.9 in secondary.

**Table 1:** Characteristics of students from the Student Mental Health Survey from Ontario, Canada by elementary (*n* = 14,665) and secondary students (*n* = 8,903)

Characteristics	Elementary Students *n* = 14,665	Secondary Students *n* = 8,903
**School-level**		
School socioeconomic status^1^: mean (SD)	86.3 (21.9)	89.4 (20.3)
School urbanity: n (%)		
Urban	12,496 (88.6%)	7333 (84.1%)
Rural	1611 (11.4%)	1388 (15.9%)
Class size: n (%)		
1–5	12 (0.1%)	255 (2.9%)
16–20	366 (2.5%)	1469 (16.5%)
21–25	5892 (40.2%)	2703 (30.4%)
26–30	7099 (48.4%)	3284 (36.9%)
31+	1226 (8.4%)	811 (9.1%)
Missing	70 (0.5%)	381 (4.3%)
**Teacher-level**		
Teacher distress: mean (SD)	4.1 (4.2)	3.9 (3.6)
**Student-level**		
Age (years): mean (SD)	12.2 (1.05)	15.5 (1.5)
Missing (n (%))	55 (0.4%)	31 (0.3%)
Gender: n (%)		
Male (reference)	6961 (47.5%)	4204 (47.2%)
Female	7635 (52.1%)	4638 (52.1%)
Missing	69 (0.5%)	61 (0.7%)
Race or cultural group: n (%)		
White (reference)	7863 (53.6%)	5385 (60.5%)
East, Southeast, South Asian	2824 (19.3%)	1389 (15.6%)
Black	792 (5.4%)	511 (5.7%)
Other/Multiracial	2902 (19.8%)	1494 (16.8%)
Missing	284 (1.9%)	124 (1.4%)
School safety: mean (SD)	16.1 (0.8)	16.3 (0.8)
Family assets: mean (SD)	-0.05 (0.3)	0.08 (0.2)
Externalizing symptoms: mean (SD)	15.8 (3.7)	16.0 (3.9)
Missing (n (%))	21 (0.1%)	8 (0.9%)
Internalizing symptoms: mean (SD)	13.3 (4.2)	14.8 (4.9)
Missing (n (%))	52 (0.35%)	5 (0.6%)

^1^Weighted median family income presented in increments of $1,000

SD: Standard Deviation; n: number

### Association between teacher distress and student internalizing symptoms and externalizing symptoms

Among elementary school students, after adjusting for age, student gender, student race or cultural group, family assets, classroom size, school socioeconomic status, and school urbanicity, for every unit increase in teacher distress, student internalizing symptoms increased by 0.02 (95% CI [< 0.01, 0.05], Table 2). Findings were similar among secondary students, with a positive association between teacher distress and internalizing symptoms (b = 0.03; 95% CI [<-0.00, 0.06]); however, the association was only statistically significant among elementary students. Similar to internalizing symptoms, for every unit increase in teacher distress, there was an increase in externalizing symptoms of 0.03 (95% CI [< 0.01, 0.05]; Table 3) among students in elementary schools.

**Table 2:** Unstandardized coefficients (b) and 95% confidence intervals (CIs) of the association between teacher distress and internalizing symptoms by elementary (*n* = 14,665) and secondary students (*n* = 8,903)

	Model 1^1^		Model 2^2^	
	Elementary b (95% CI); *p*-value	Secondary b (95% CI); *p*-value	Elementary b (95% CI); *p*-value	Secondary b (95% CI); *p*-value
Teacher distress	0.02 (< 0.01, 0.04); *p* = 0.03	0.03 (<-0.01, 0.06); *p* = 0.12	0.08 (<-0.01, 0.16); *p* = 0.06	0.02 (-0.12, 0.16); *p* = 0.77
Student age (years)	0.34 (0.27, 0.41); *p* < 0.001	0.25 (0.17, 0.33); *p* < 0.001	0.27 (0.21, 0.34); *p* < 0.001	0.35 (0.27, 0.43); *p* < 0.001
Student gender				
Female	1.43 (1.29, 1.56); *p* < 0.001	2.62 (2.41, 2.82); *p* < 0.001	1.43 (1.31, 1.56); *p* < 0.001	2.46 (2.26, 2.67); *p* < 0.001
Male (reference)				
Race or cultural group				
White (reference)				
East, Southeast, South Asian	-0.17 (-0.38, 0.04); *p* = 0.12	-0.17 (-0.50, 0.16); *p* = 0.31	-0.13 (-0.32, 0.07); *p* = 0.02	-0.33 (-0.65, -0.02); *p* = 0.04
Black	-0.61 -0.93, -0.29); *p* < 0.001	-0.57 (-1.02, -0.12); *p* = 0.01	-0.76 (-1.06, -0.45); *p* < 0.00	-0.99 (-1.43, -0.56); *p* < 0.001
Other/Multiracial	0.22 (0.06, 0.41); *p* = 0.02	0.48 (0.20, 0.76); *p* < 0.001	0.007 (-0.17, 0.18); *p* = 0.60	0.29 (-0.02, 0.56); *p* = 0.04
Family assets	-0.36 (-0.86, 0.14); *p* = 0.16	-0.40 (-1.41, 0.60); *p* = 0.43	-0.08 (-0.39, 0.22); *p* = 0.54	-0.16 (-1.16, 0.68); *p* = 0.74
Classroom size				
1–5	0.29 (-2.11, 2.67); *p* = 0.81	0.14 (-0.52, 0.80); *p* = 0.68	0.73 (-1.44, 2.91); *p* = 0.47	-0.13 (-0.76, 0.50); *p* = 0.97
16–20	0.17 (-0.34, 0.68); *p* = 0.51	0.32 (-0.03, 0.66); *p* = 0.07	0.38 (-0.11, 0.86); *p* = 0.16	0.20 (-0.13, 0.53); *p* = 0.23
21–25	0.12 (-0.05, 0.30); *p* = 0.17	0.20 (-0.09, 0.48); *p* = 0.17	0.01 (-0.15, 0.18); *p* = 0.69	-0.09 (-0.18, 0.36); *p* = 0.52
26–30 (reference)				
31+	-0.14 (-0.45, 0.17); *p* = 0.39	0.23 (-0.21, 0.67); *p* = 0.31	-0.12 (-0.41, 0.18); *p* = 0.27	0.26 (-0.16, 0.68); *p* = 0.22
School SES^2^	-0.01 (-0.01, < 0.01); *p* = 0.10	< 0.00 (-0.01, 0.01); *p* = 0.94	-0.002 (-0.01, 0.01); *p* = 0.71	< 0.00 (<-0.01, 0.02); *p* = 0.45
School urbanity	-0.05 (-0.36, 0.25); *p* = 0.74	0.11 (-0.32, 0.54); *p* = 0.62	-0.09 (-0.39, 0.22); *p* = 0.83	0.08 (-0.33, 0.48); *p* = 0.71
School safety			-0.44 (-0.47, -0.41); *p* < 0.001	-0.40 (-0.44, -0.35); *p* < 0.001
School safety*teacher distress			-0.01 (-0.01, <-0.00); *p* = 0.06	< 0.01 (<-0.01, 0.01); *p* = 0.97

^1^Model adjusted for student age, student gender, student race or cultural group, family assets, classroom size, school socioeconomic status, school urbanicity

^2^Model adjusted for student age, student gender, student race or cultural group, family assets, classroom size, school socioeconomic status, and school urbanicity with the addition of the main effect for school safety and an interaction term for school safety and teacher distress

CI: confidence interval; b: unstandardized coefficient; SES: socioeconomic status

**Table 3:** Unstandardized coefficients (b) and 95% confidence intervals (CIs) of the association between teacher distress and externalizing symptoms by elementary (*n* = 14,665) and secondary students (*n* = 8,903)

	Model 1^1^		Model 2^2^	
	Elementary b (95% CI); *p*-value	Secondary b (95% CI); *p*-value	Elementary b (95% CI); *p*-value	Secondary b (95% CI); *p*-value
Teacher distress	0.03 (< 0.01, 0.05); *p* < 0.001	-<0.01 (-0.03, 0.03); *p* = 0.99	0.12 (0.05, 0.18); *p* < 0.001	-0.06 (-0.18, 0.06); *p* = 0.30
Student age (years)	0.27 (0.20, 0.33); *p* < 0.001	-0.01 (-0.07, 0.08); *p* = 0.88	0.30 (0.24, 0.36); *p* < 0.001	< 0.07 (<-0.01, 0.14); *p* = 0.06
Student gender				
Female	-0.88 (-1.00, -0.76); *p* < 0.001	-0.90 (-1.07, -0.73); *p* < 0.001	-0.88 (-0.99, -0.76); *p* < 0.001	-1.01 (-1.18, -0.85); *p* < 0.001
Male (reference)				
Race or cultural group				
White (reference)				
East, Southeast, South Asian	-0.19 (-0.38, -0.01); *p* = 0.04	0.06 (-0.22, 0.33); *p* = 0.70	-0.27 (-0.44, -0.09); *p* < 0.001	0.07 (-0.39, 0.20); *p* = 0.60
Black	0.56 (0.28, 0.84); *p* < 0.001	0.93 (0.56, 1.30); *p* < 0.001	0.45 (0.18, 0.73); *p* < 0.001	0.64 (0.28, 1.01); *p* < 0.001
Other/Multiracial	0.43 (0.27, 0.59); *p* < 0.001	0.53 (0.30, 0.76); *p* < 0.001	0.31 (0.16, 0.47); *p* < 0.001	0.41 (0.18, 0.64); *p* < 0.001
Family assets	-0.48 (-1.00, -0.04); *p* = 0.03	0.52 (-0.39, 1.44); *p* = 0.26	-0.33 (-0.80, 0.15); *p* = 0.17	0.68 (-0.14, 1.50); *p* = 0.10
Classroom size				
1–5	2.31 (0.15, 4.47); *p* = 0.07	1.02 (0.38, 1.44); *p* < 0.001	2.66 (0.58, 4.74); *p* = 0.01	0.85 (0.25, 1.45); *p* = 0.01
16–20	0.01 (-0.44, 0.47); *p* = 0.04	0.22 (-0.39, 1.65); *p* = 0.22	0.11 (-0.31, 0.54); *p* = 0.61	0.14 (-0.19, 0.46); *p* = 0.42
21–25	0.12 (-0.04, 0.28); *p* = 0.96	0.27 (-0.02, 0.56); *p* = 0.08	0.07 (-0.08, 0.22); *p* = 0.32	0.19 (-0.09, 0.46); *p* = 0.18
26–30 (reference)				
31+	-0.09 (-0.37, 0.20); *p* = 0.55	-0.13 (-0.60, 0.39); *p* = 0.58	-0.07 (-0.34, 0.18); *p* = 0.55	-0.11 (-0.55, 0.32); *p* = 0.61
School SES^2^	-0.01 (-0.01, < 0.01); *p* = 0.06	-0.01 (-0.02, < 0.01); *p* = 0.09	<-0.01 (-0.01, < 0.01); *p* = 0.29	-0.01 (-0.02, < 0.01); *p* = 0.43
School urbanity	0.26 (-0.05, 0.58); *p* = 0.10	0.40 (< 0.01, 0.79); *p* = 0.05	0.27 (-0.02, 0.56); *p* = 0.06	0.38 (-0.03, 0.73); *p* = 0.03
School safety			-0.27 (-0.29, 0.-24); *p* < 0.001	-0.27 (-0.31, -0.23); *p* < 0.001
School safety*teacher distress			<-0.01 (-0.01, <-0.01); *p* < 0.001	< 0.01 (<-0.01, 0.01); *p* = 0.32

^1^Model adjusted for student age, student gender, student race or cultural group, family assets, classroom size, school socioeconomic status, school urbanicity

^2^Model adjusted for student age, student gender, student race or cultural group, family assets, classroom size, school socioeconomic status, and school urbanicity with the addition of the main effect for school safety and an interaction term for school safety and teacher distress

CI: confidence interval; b: unstandardized coefficient; SES: socioeconomic status

### Modification by school safety

**Fig. 2:**
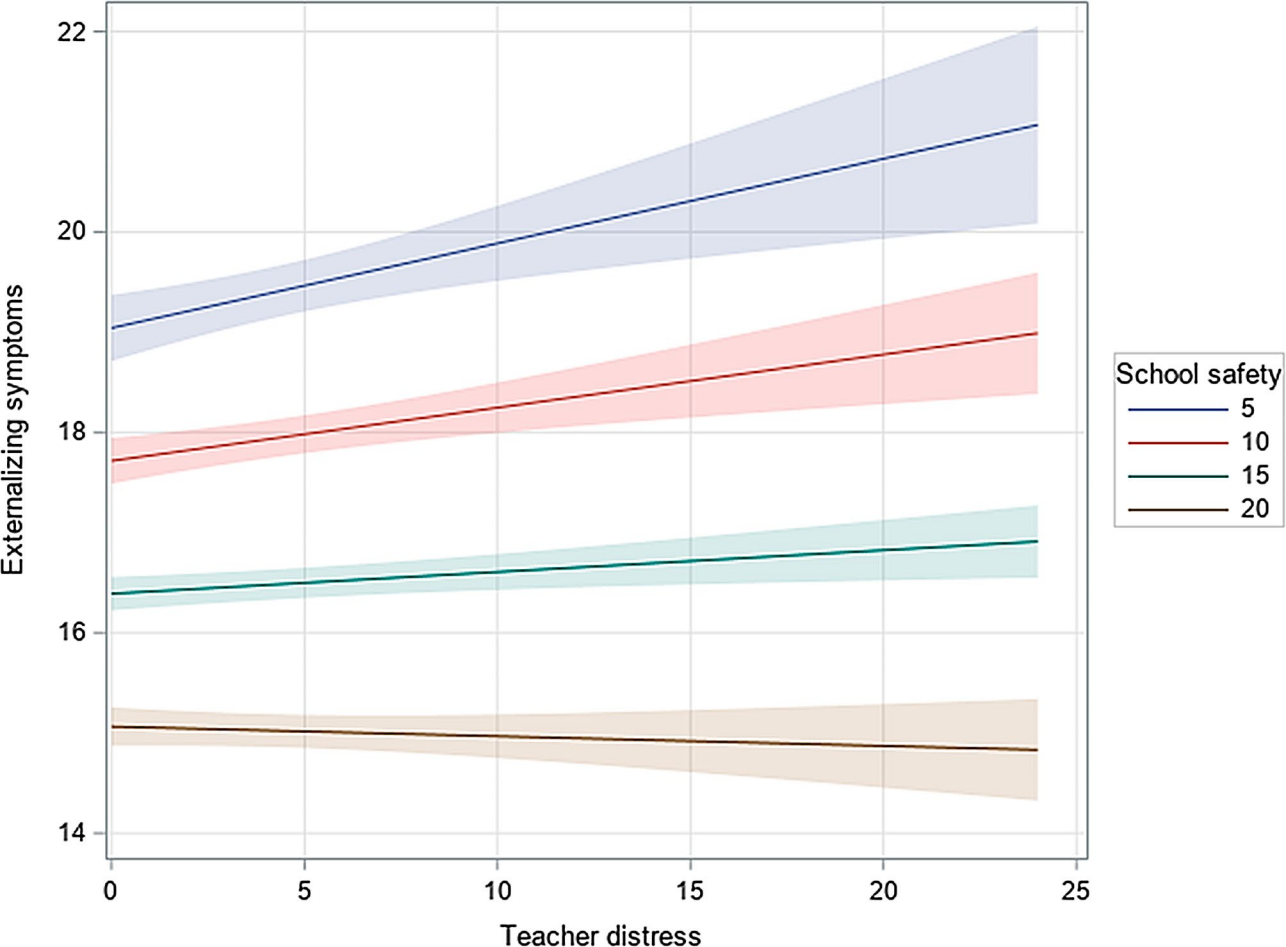
School safety moderates the association between teacher distress and externalizing symptoms among students in elementary school (*n* = 14,665). Caption: School safety score ranged from 5–20, whereby 5 represented the lowest safety, and 20 represented the highest perceived safety. Simple slopes for school safety were analyzed in increments of 5 (5, 10, 15, 20). When analyzing simple slopes, the association indicated that for every unit increase in teacher distress, there was a significant increase in externalizing symptoms, but only when perceived school safety was lower. When school safety was high (e.g., school safety = 20), the association between teacher distress and externalizing symptoms was not statistically significant. Shaded bars represent 95% confidence intervals

Effect modification by student-reported school safety was examined by adding interaction terms with teacher distress for internalizing and externalizing symptoms (Tables 2 and 3). The main effects for school safety for both outcomes across elementary and secondary students were statistically significant (internalizing symptoms, grades 6–8: b = -0.44, 95% CI [-0.47, -0.41]; internalizing symptoms, secondary: b=-0.40, 95% CI [-0.44, -0.35]; externalizing symptoms, elementary: b = -0.27, 95% CI [-0.29, -0.24]; externalizing symptoms, secondary: b = -0.27, 95% CI [-0.31, -0.23]). There was little evidence of an interaction between school safety and teacher distress for internalizing symptoms (b = 0.01, 95% CI [-0.01, <-0.001]; *p* = 0.06), but the interaction was significant for externalizing symptoms (b = <-0.01, 95% CI [-0.01, <-0.01]; *p* < 0.001) among elementary school students only. When analyzing simple slopes, the association indicated that for every unit increase in teacher distress, there was a significant increase in externalizing symptoms but only when perceived school safety was lower, e.g., when school safety was 5 (b = 0.08; 95% CI: 0.04, 0.13), 10 (b = 0.05; 95% CI [0.02, 0.08], and 15 (b = 0.02; 95% CI [0.01, 0.04] (Fig. 2). When perceptions of school safety were high (e.g., school safety = 20), the unstandardized coefficient for the simple slope was − 0.01 and not statistically significant (95% CI [-0.03, 0.01]; *p* = 0.43).

## Discussion

This study found that teacher distress was positively associated with student internalizing and externalizing symptoms among elementary students only. This association was evident after adjusting for comprehensive sociodemographic and economic covariates. We also found that student perceptions of school safety were negatively associated with internalizing and externalizing symptoms among both elementary and secondary students. Moreover, school safety moderated the association between teacher distress and externalizing symptoms among elementary students, whereby the positive association was magnified among students reporting lower levels of school safety.

Although standardized coefficients (see Appendix) were relatively small for all associations between teacher distress and student mental health outcomes, it is imperative to recognize the multilevel nature of this association, meaning that one single teacher may influence the mental health and well-being of all students within their classroom. Only one study (Harding et al., 2019), to our knowledge, had a similar objective to the current study and found that students who had teachers with better well-being had lower psychological distress (standardized effect = -0.10; 95% CI [-0.16, -0.04] and better overall well-being (standardized effect = 0.07; 95% CI [0.02, 0.12]. Similar to the present study, Harding and colleagues (2019) reported small standardized coefficients and inferred that the small magnitude of effects may have been related to low variance in teacher distress in their sample [2]. They also noted that while findings may seem small at an individual level, at a broader population level, these findings may still have meaningful impacts, which has also been noted in other research [33]. The explanations for the small effect sizes may apply to the current study’s findings. Like Harding et al. (2019), we also observed low variance in teacher distress in our sample, with mean scores of approximately four on a 24-point scale. Additionally, applying the emotional contagion theory to a classroom setting where many students are engaged in multiple interactions across the day with the teacher and with each other may lead to small individual-level effect sizes but meaningful impacts at the population level. Other research that generally focused on teacher burnout or well-being and student outcomes found somewhat similar results, where teacher burnout or poor well-being was associated with worse student performance academically and socially, lack of motivation, higher reports of stress, and decreased perception of social support [2, 5, 14, 15, 17].

Consistent with prior literature, low perceived school safety was found to be positively associated with student internalizing and externalizing symptoms, meaning students who had lower perceptions of school safety reported higher levels of mental health symptoms [19]. While previous studies have examined associations between school safety and student mental health and well-being [7, 18], few have examined the moderating effects on associations between teacher and student mental health outcomes. In the present study, we found that lower perceptions of safety magnified the association between teacher distress and student externalizing symptoms. This is an important finding, as schools and school boards may focus on targeting safety within schools to mitigate risk factors associated with poor student mental health. At a time when teacher distress is exceptionally high as a result of the COVID-19 pandemic [34], these findings speak to the importance of taking a multilevel and whole-school approach to student mental health prevention. It is also important to consider both existing and new interventions or factors that will maintain or promote the well-being of teachers as this will simultaneously have beneficial outcomes for students.

Our results confirm the importance of considering different factors that may influence student mental health, specifically interpersonal factors within the school environment, as suggested by Townsend and Foster [21]. Although our study did not explore the exact mechanisms underlying this association, social contagion processes between teachers and students are a possible avenue worthy of further exploration, as suggested by the emotional contagion theory [22]. Lazarus and Folkman’s Transactional Model of Stress and Coping [35] states that stress may be the result of an appraisal of an external stressor that triggers an emotional response. This model is relevant when considering teacher-student relationships, as exposure to chronic stress within the school or classroom environment may impact the well-being of both teachers and students. Future research is needed to explore potential, intermediary mechanisms underlying the association between teacher and student mental health, in order to inform prevention and intervention strategies. As our results suggest, it is essential to consider interventions addressing the mental health needs of both students and teachers concurrently and over time. It will also be important for future research to examine how individual characteristics of teachers, including age, sex, or years of teaching, may co-occur with differing levels of teacher distress. Understanding how these simultaneously occur may help to identify specific subgroups of teachers who can benefit from additional supports.

### Strengths and limitations

The SMHS is a unique and comprehensive study that used a stratified sampling approach to enlist a representative sample of schools [25]. In addition, the sample size for this study was large, and the characteristics of the included sample of schools was representative [25]. Another strength of this study is that the psychometric properties of the scales used to measure teacher distress and student mental health outcomes have been tested, and have been found to have good reliability and validity [26]. In addition to the psychometric properties of the measures used to collect data in the SMHS, there was in-depth coverage and measurement of important school, classroom, and student-level sociodemographic and economic characteristics, permitting statistical adjustment of these potential confounding influences on teacher and student mental health outcomes. A potential limitation of this study is related to the cross-sectional nature of the study design, as this does not allow for assessments of temporality. Although we assessed the impact of teacher distress on student mental health outcomes, it is possible and likely that this association is reciprocal due to emotional cross-cover or contagion in a classroom and the co-construction of experiences between teachers and students [36]. For example, prior work has suggested that teacher distress may be partly caused by conflicts between perceived responsibility to support students in difficulty and perceived lack of resources to do so [37]. Future research can employ longitudinal methods to understand these associations’ temporal nature and directionality. Longitudinal research will help pinpoint causal mechanisms to target interventions that address teacher and student distress. Another limitation is that it was difficult to assess secondary students’ complete exposure to teacher distress, as students in secondary schools typically have multiple teachers throughout a school day and school year. Future research may consider collecting more detailed information across all secondary students’ teachers and/or creating a composite measure at the school-contextual level. Finally, multiple imputations to account for missing data were not run due to our data’s multilevel and cross-informant nature. Currently there is no consensus on how to apply multiple imputations for highly clustered, multilevel data, given the complexity of handling missing data when accounting for random effects and interaction terms [38, 39]. Simulation studies have been conducted to explore the use of multiple imputations when using multilevel data; however, there are several limitations, as effect sizes, sample sizes, and constant values are arbitrarily decided and may not be relevant for more complex research questions [38].

### Implications

The findings from this study can be used to inform the development of mental health prevention and intervention strategies designed to support the mental health needs of both teachers and students. Continued research is needed to explore the underlying mechanisms associated with the inter-relationships between teacher and students’ mental health and resilience so that targeted and tailored strategies are developed [40].

## Conclusions

The learnings from this research highlight the importance of supporting and effectively addressing feelings of distress among teachers. Doing so will benefit not only teachers’ health and well-being but also their students. Finally, given the increasing levels of student mental ill-health identifying risk factors, including within the school environment, are important to inform the development targeted, prevention efforts.

## Electronic supplementary material

Below is the link to the electronic supplementary material.

Supplementary Material 1

## Data Availability

The datasets used and/or analysed during the current study are available from the corresponding author upon reasonable request.

## Abbreviations

CI: Confidence Interval
DSM: Diagnostic and Statistical Manual of Mental Disorders
OCHS: Ontario Child Health Study
OCHS-EBS: Ontario Child Health Study Emotional Behavioural Scales
SMHS: School Mental Health Survey
SD: Standard Deviation
