# Supplementary material for: The association between teacher distress and student mental health outcomes: a cross-sectional study using data from the school mental health survey
Source: BMC Psychol. 2024 Oct 23;12:583. doi: 10.1186/s40359-024-02071-3 (PMC11520149; doi:10.1186/s40359-024-02071-3)
Supplement: Supplementary file 1 — Supplementary Material 1 [file 40359_2024_2071_MOESM1_ESM.docx]

**Table A1.** Characteristics of teachers from the Student Mental Health Survey from Ontario, Canada by elementary (n=882) and secondary school (n=596).

|  | **Elementary Teachers**  **n=882** | **Secondary Teachers**  **n=596** |
| --- | --- | --- |
| Gender: n (%)  Female  Male  Missing | 565 (64.1%)  308 (34.9%)  9 (1.0%) | 351 (58.9%)  245 (41.1%)  0 |
| Race or Cultural Group: n (%)  White  Other  Missing | 766 (86.8%)  98 (11.1%)  18 (2.0%) | 522 (87.5%)  61 (10.2%)  13 (2.2%) |
| Born in Canada: n (%)  Yes  No  Missing | 772 (87.5%)  100 (11.3%)  10 (1.1%) | 523 (87.8%)  71 (11.9%)  2 (0.3%) |
| Years of experience working as a teacher: n (%)  3 years or less  3-5 years  6-10 years  Over 10 years  Missing | 67 (7.6%)  77 (8.7%)  201 (22.8%)  492 (55.8%)  45 (5.1%) | 19 (3.2%)  44 (7.4%)  131 (22.0%)  376 (63.1%)  26 (4.4%) |

**Table A2.** Standardized coefficients^1^ (β) and 95% confidence intervals (CIs) of the association between teacher distress and internalizing symptoms by elementary (n=14,665) and secondary students (n=8,903)

|  | Model 1^2^ | | Model 2^3^ | |
| --- | --- | --- | --- | --- |
|  | Elementary  β (95% CI); p-value | Secondary  β (95% CI); p-value | Elementary  β (95% CI); p-value | Secondary  β (95% CI); p-value |
| Teacher distress | 0.02 (<0.0, 0.04); p=0.03 | 0.02 (-0.01, 0.05); p=0.12 | 0.00 (-0.01, 0.02); p=0.66 | 0.02 (-0.01, 0.04); p=0.25 |
| Student age (years) | 0.07 (0.06, 0.09); p=<0.00 | 0.05 (0.04, 0.07); p=<0.00 | 0.09 (0.07, 0.10); p<0.00 | 0.08 (0.06, 0.09); p=<0.00 |
| Student gender  Female  Male (reference) | 0.31 (0.28, 0.34); p=<0.00 | 0.57 (0.53, 0.62); p=<0.00 | 0.31 (0.28, 0.34); p<0.00 | 0.54 (0.50, 0.58); p=<0.00 |
| Race/ethnicity  White (reference)  East, Southeast, South Asian  Black  Other/Multiracial | -0.04 (-0.08, 0.01); p=0.11  -0.13 (-0.20. -0.06); p=<0.00  0.05 (0.01, 0.09); p=0.02 | -0.04 (-0.11, 0.03); p=0.31  -0.12 (-0.22, -0.03); p=0.01  0.11 (0.05, 0.17); p=<0.00 | -0.06 (-0.10, -0.02); p=0.01  -0.16 (-0.23, -0.10); p<0.00  0.01 (-0.03, 0.05); p=0.69 | -0.07 (-0.14, -0.00); p=0.04  -0.22 (-0.31, -0.12); p=<0.00  0.06 (0.00, 0.12); p=0.04 |
| Family assets | -0.00 (<-0.00, 0.00); p=0.10 | 0.00 (-0.00, 0.00); p=0.93 | -0.00 (-0.00, 0.00); p=0.72 | 0.00 (-0.00, 0.12); p=0.04 |
| Classroom size  1-5  16-20  21-25  26-30 (reference)  31+ | 0.06 (-0.46, 0.59); p=0.81  0.04 (-0.07, 0.15); p=0.51  0.03 (-0.01, 0.07); p=0.17  -0.03 (-0.10, 0.04); p=0.39 | 0.03 (-0.00, 0.00); p=0.93  0.07 (-0.11, 0.18); p=0.07  0.04 (-0.02, 0.11); p=0.17  0.05 (-0.05 0.15); p=0.31 | 0.19 (-0.30, 0.69); p=0.45  0.08 (-0.03, 0.18); p=0.16  0.02 (-0.02, 0.05) p=0.41  -0.02 (-0.09, 0.04); p=0.48 | -0.03 (-0.17, 0.11); p=0.69  0.04 (-0.03, 0.12); p=0.11  0.02 (-0.03, 0.12); p=0.23  0.06 (-0.03, 0.15); p=0.22 |
| School SES^2^ | -0.08 (-0.05, 0.01 p=0.16 | -0.03 (-0.09, 0.04); p=0.43 | -0.00 (-0.03, 0.03); p=0.77 | -0.01 (-0.07, 0.05); p=0.74 |
| School urbanity | -0.01 (-0.08, 0.06); p=0.74 | 0.02 (-0.07, 0.12); p=0.62 | -0.01 (0.07, 0.05); p=0.74 | 0.02 (-0.07, 0.11); p=0.71 |
| School safety |  |  | -0.32 (-0.33, -0.30); p<0.00 | -0.28 (-0.30, -0.26); p=<0.00 |
| School safety*teacher distress |  |  | -0.01 (-0.03, 0.00); p=0.08 | -0.00 (-0.02, 0.02); p=0.97 |

1. Variables were standardized using the following formula: $B=\frac{s_{x}}{s_{y}}$ where s_x_ and s_y_ are the estimated standard deviations of x and y, respectively
2. Model adjusted for student age, student gender, student race/ethnicity, family assets, classroom size, school socioeconomic status, school urbanicity
3. Model adjusted for student age, student gender, student race/ethnicity, family assets, classroom size, school socioeconomic status, school urbanicity with the addition of the main effect for school safety and interaction term for school safety and teacher distress

CI: confidence interval; β: beta coefficient; SES: socioeconomic status

**Table A3.** Standardized coefficients^1^ (β) and 95% confidence intervals (CIs) of the association between teacher distress and externalizing symptoms by elementary (n=14,665) and secondary students (n=8,903)

|  | Model 1^2^ | | Model 2^3^ | |
| --- | --- | --- | --- | --- |
|  | Elementary  β (95% CI); p-value | Secondary  β (95% CI); p-value | Elementary  β (95% CI); p-value | Secondary  β (95% CI); p-value |
| Teacher distress | 0.03 (0.01, 0.05); p=0.00 | -0.00 (-0.03, 0.03); p=0.99 | 0.01 (-0.00, 0.03); p=0.09 | -0.00 (-0.04, 0.03); p=0.71 |
| Student age (years) | 0.07 (0.05, 0.09); p<0.00 | 0.00 (-0.01, 0.02); p=0.88 | 0.08 (0.06, 0.09); p<0.00 | 0.02 (-0.00, 0.04); p=0.06 |
| Student gender  Female  Male (reference) | -0.23 (-0.26, -0.20); p<0.00 | -0.24 (-0.28, -0.19); p<0.00 | -0.23 (-0.26, -0.20); p<0.00 | -0.27 (-0.31, -0.22); p<0.00 |
| Race/ethnicity  White (reference)  East, Southeast, South Asian  Black  Other/Multiracial | -0.05 (-0.10, -0.00); p=0.04  0.15 (0.07, 0.22); p=0.00  0.11 (0.07, 0.16); p<0.00 | - 1. (-0.06, 0.09); p=0.70   0.24 (0.15, 0.34); p<0.00  0.14 (0.08, 0.20); p<0.00 | -0.07 (-0.12, -0.02); p=0.00  0.12 (0.05, 0.19); p=0.00  0.08 (0.04, 0.12); p<0.00 | -0.02 (-0.09, 0.05); p=0.60  0.17 (0.07, 0.27); p=0.00  0.11 (0.05, 0.17); p=0.00 |
| Family assets | -0.00 (-0.00, 0.00); p=0.06 | -0.00 (-0.01, 0.00); p=0.09 | -0.00 (-0.00, 0.00); p=0.28 | -0.00 (-0.00, 0.00); p=0.14 |
| Classroom size  1-5  16-20  21-25  26-30 (reference)  31+ | 0.61 (0.04, 1.18); p=0.04  0.03 (-0.12, 0.12); p=0.96  0.03 (-0.01, 0.07); p=0.14  -0.02 (-0.10, 0.05); p=0.55 | 0.27 (0.10, 0.43); p=0.09  0.06 (-0.03, 0.15); p=0.00  0.07 (-0.01, 0.15); p=0.07  -0.03 (-0.16, 0.09); p=0.58 | 0.70 (0.15, 1.24); p=0.01  0.03 (-0.08, 0.14); p=0.61  0.02 (-0.01, 0.06); p=0.32  -0.02 (-0.09, 0.04); p=0.55 | 0.22 (0.06, 0.38); p=0.01  0.04 (-0.05, 0.12); p=0.42  0.05 (-0.02, 0.12); p=0.18  -0.03 (-0.14, 0.08); p=0.61 |
| School SES^2^ | -0.04 (-0.07, 0.00); p=0.07 | 0.04 (-0.03, 0.11); p=0.26 | -0.02 (-0.06, 0.01); p=0.17 | 0.05 (-0.01, 0.11); p=0.10 |
| School urbanity | 0.07 (-0.01, 0.15); p=0.10 | 0.10 (0.00, 0.21); p=0.05 | 0.07 (-0.00, 0.15); p=0.06 | 0.10 (0.07, 0.19); p=0.03 |
| School safety |  |  | -0.24 (-0.36, -0.23); p<0.00 | -0.21 (-0.24, -0.19); p<0.00 |
| School safety*teacher distress |  |  | -0.02 (-0.04, -0.01); p=0.00 | 0.01 (-0.01, 0.04); p=0.32 |

1. Variables were standardized using the following formula: $B=\frac{s_{x}}{s_{y}}$ where s_x_ and s_y_ are the estimated standard deviations of x and y, respectively
2. Model adjusted for student age, student gender, student race/ethnicity, family assets, classroom size, school socioeconomic status, school urbanicity
3. Model adjusted for student age, student gender, student race/ethnicity, family assets, classroom size, school socioeconomic status, school urbanicity with the addition of the main effect for school safety and interaction term for school safety and teacher distress

CI: confidence interval; β: beta coefficient; SES: socioeconomic status
